# Extract from Bartlett’s Discourse on Hippocrates

**Published:** 1853-04

**Authors:** 


*Extract from Bartlett's Discourse on Hippocrates.*—And now, leaving the sterile island of Thasos, let us follow the young physician to another sick chamber—to a scene of domestic life, still further illustrative of that remote and wonderful period, with which we are concerned.

The time is a year or two later—it is the house of Pericles that we enter, and stand by the death-bed of the great and venerable Archon. Every thing in the spacious apartment indicates the pervading presence—not of obtrusive grandeur, or of showy and ostentatious wealth—but of stately elegance, and of high, various, many-sided luxury, culture and refinement. Philosophy, letters and art breathe in the quiet atmosphere of the room; and the taste of Aspasia sheds an Asiatic grace over its furnishing and its decorations. In one corner stands a statue of Minerva from the chisel of Phidias; and the walls are covered with pictures, fresh from the pencils of Panænus and Polygnotus, illustrating the legendary and historic glories of Greece. There might have been seen Thêseus, bearing off from the field of victory, on the banks of the Thermódon the masculine and magnificent queen of the Amazons—half willing, perhaps, to be the captive of such a victor; Jason, in his good ship Argo, with his fifty selectest heroes, convoyed by the queen of love, and the awful Hêre, and Apollo, winds his various and adventurous voyage, crowded with poetic imagery and romantic incident, and brings back the golden fleece from Colchis; Helen, at her loom, is weaving into her "golden web" the story of the Trojan wars; the chaste Penelope, by the light of her midnight lamp, undoes the delusive labors of the day; Ulysses, returned from his long wanderings, surveys once more, with boyish pride and delight, the dear old bow, which no arm but his could bend.

The central figure on that old historic canvas that I have endeavored to unroll before you, is that of the dying statesman. Raised, and resting in solemn and august serenity upon its last pillow, lies that head of Olympian grandeur, which—I may say it without presumption—after the lapse of twenty-three centuries, now finds, for the first time, its fitting representative and likeness—as the character and career of the great Athenian find their counterparts also—in that illustrious orator and statesman, who now walks in solitary majesty amongst us—the pride, the strength, the glory of the Republic—the Pericles of our Athens—whose Acropolis is the Constitution of his country—whose Propylæa are the freedom and the federation of the States.

Added to the calamities of that long and disastrous internecine struggle between the two rival cities of Greece, which had just begun, Athens was now afflicted with that terrible visitation of the plague, the history of which has been left to us by Thucydides; and Pericles was sinking under a protracted and wearing fever—the result of an attack of the disease.

His long and glorious life is about to close. He had been, for more than an entire generation—if never the first Archon, and not always the most popular—by common consent the most eminent citizen, statesman, and orator, of the Republic—the great defender of her Constitution—the champion of her freedom and her rights—the upholder and the magnifier of her renown. Political rivals, diappointed partizans, and a few malignant personal enemies, and professional libellers and satirists, had been hostile to his carreer, and had endeavored to blacken his fair fame ; but his strong and unshaken democratic faith —his far-seeing sagacity—his firmness and moderation—his enlarged, liberal, humanizing, conservative, and pacific policy—his moral courage and independence, and his high public probity, had triumphed over them all; and although by braving the prejudices of his friends and supporters, in his devotion to the general weal, he had gathered over his declining sun some clouds of public disfavor—the sense of justice, and the feeling of gratitude, in the minds of his countrymen were quick to return—the clouds were already scattered, or they served only to deepen and reflect the setting splendor which, for a moment, they had intercepted and obscured.

Many of his near personal friends and relatives had already fallen victims to the pestilence. Both his sons had perished, and the young Pericles—the child of Aspasia—had been sent away, with his mother, for safety, into Thessaly. Phidias, and his old teacher Anaxagoras, his

"Guide, philosopher, and friend,"

had died a little while before the breaking out of the epidemic. Those who were left had now gathered around the bed af the dying Archon, to receive the rich legacy of his parting words, and to pay to him the last solemn and kindly offices of life.

Not often in the world's history has there met together a more august and illustrious company. These are a few of tho e whom we are able to recognize amongst them : Resting his head on the shoulder of Socrates, and sobbing aloud in unrestrained and passionate sorrow, leans the wild and reckless Alcibiades—just in the first bloom of that resplendent personal beauty which made him seem to the eyes, even of the Greeks, more like the radiant apparition of a young Apollo, than any form of a mere earthly mould—subdued, for the first time in his life, and probably for the last—by the spectacle before him, of his dying relative and guardian—to reverence, tenderness and truth. Sophocles, his old companion in arms, is there ; and near him, in his coarse mantle, and with unsandalled feet, may have stood a grandson of Aristides, still poor with the honorable poverty of his great ancestor.

Conspicuous amidst this group of generals, admirals, statesmen, orators, artists, poets, and philosophers—in rank and fortune, in social position, in reputation, in learning, culture and refinement, their equal and associate, sits the young physician of Cos. Already had his rising fame reached Athens, and when the city, overcrowded with the inhabitants of Attica, driven from their home by the armies of Sparta, was smitten with the pestilence, he was summoned from his island home in the Ægean, to stay, if he could, the march of the destroying angel, and to succor with his skill those who had fallen under the shadow of its wings.

